# Associations between neck circumference and markers of dysglycemia, non-alcoholic fatty liver disease, and dysmetabolism independent of Body Mass Index in an Emirati population

**DOI:** 10.3389/fendo.2022.929724

**Published:** 2022-09-06

**Authors:** Esphie Grace Fodra Fojas, Adam John Buckley, Nader Lessan

**Affiliations:** Research Institute, Imperial College London Diabetes Centre, Abu Dhabi, United Arab Emirates

**Keywords:** neck circumference, obesity, dysglycemia, NAFLD, MetS

## Abstract

**Aim:**

Neck circumference (NC) is quick and easy to measure and may be a useful surrogate marker for body composition. We investigated NC as a potential marker of dysglycemia, MetS, and NAFLD.

**Methods:**

674 individuals were recruited at the Imperial College London Diabetes Centre in a study of sleep apnea prevalence. Of these, 547 (Age 46 ± 11.4 years, Body Mass Index (BMI) 31 ± 6 kg/m^2^, 279 (51%) female, 113 normal glucose tolerance (NGT), 108 Prediabetes, 326 Type 2 diabetes (T2DM)) met all inclusion criteria for analysis. NC was measured at the thyroid cartilage, and collar size was recorded. Analysis was performed using univariate and multivariate linear regression.

**Results:**

Adjusted for BMI, sex, and age, NC was 0.65 ± 0.3 cm greater in prediabetes (p = 0.0331), and 1.07 ± 0.28 cm greater in T2DM, compared with NGT (p = 0.0002). Adjusting for BMI, sex, and glycemic status, 1-cm increase in NC was associated with a 1.04 ± 1.01 U/L (p <0.0001) increase in ALT and, additionally, correcting for statin use, a 0.03 ± 0.01 mmol/L reduction in HDL (p <0.0001) and a 0.1 ± 0.02 increase in TC : HDL. A 1 cm increase in NC was associated with a 1.15 ± 1.02% (p <0.0001) increase in 10-year AHA cardiovascular risk in individuals over 40 years old and a 0.16 ± 0.02 (p <0.0001) increase in NAFLD fibrosis score. The neck circumference was associated with the hazard of new onset of deranged ALT adjusted for age, sex, glycemic status, and BMI (hazard ratio 1.076 (95% CI 1.015–1.14, p = 0.0131) and with the incidence of Fatty Liver Index associated with high probability of NAFLD (hazard ratio 1.153 (95% CI 1.019–1.304), p = 0.0239).

**Conclusion:**

NC is associated with dysglycemia, components of the MetS, and factors predictive of NAFLD, but does not appear to independently predict subsequent progression to high risk of liver fibrosis in this predominantly diabetic population.

## Introduction

The prevalence of obesity is increasing worldwide, as are comorbidities including Type 2 diabetes mellitus (T2DM) and cardiovascular disease (CVD). The body mass index (BMI) criterion is endorsed by both the National Institutes of Health and the World Health Organization for defining and classifying obesity (1). However, the use of BMI as an index of adiposity has been debated, primarily because it does not reflect body fat distribution (2–4). Evidence from observational studies suggests that body fat distribution more accurately predicts cardiovascular outcomes in obese individuals (5).

Alternatives to BMI used to assess body composition include skinfold thickness, waist circumference (WC), hip circumference (HC), body adiposity index (BAI), and waist to hip ratio (WHR) (6, 7). WHR has been shown to be more strongly associated with CVD events and T2DM mortality compared with WC and BMI (8). Among the Emiratis, WHR has been reported to be a better predictor of T2DM than BMI (9). WHR measurement can be time-consuming and more prone to errors, however. Relevant anatomical landmarks can also be obscured in obese individuals.

The neck circumference (NC) has also been proposed as a measure of body composition (10, 11). NC is recognized as a risk factor for obstructive sleep apnea, which is itself associated with CVD, cardiac arrhythmias, and heart failure. NC can be measured without requiring the patient to undress, and NC landmarks may be better preserved compared with those used to measure WC in the context of obesity. NC does not vary with food intake and has been associated with central adiposity (12).

It has been suggested that NC acts as a marker of risk for the metabolic syndrome (MetS) as well as its individual features in both adult and pediatric populations (13–16), although a direct association of MetS diagnosis with NC was not demonstrated in a recent meta-analysis (17). The components and features of MetS, including dysglycemia and non-alcoholic fatty liver disease (NAFLD), have also been directly linked with NC (18–20), although population-specific cut-off points on stratified categories, such as sex and age, may be needed for reliable association of NC with MetS (21–23).

NC therefore shows some promise in assessing metabolic risk and screening for conditions associated with diabetes and obesity. Here we investigated NC as a potential marker of MetS, dysglycemia, and NAFLD in an Emirati outpatient cohort of people with normoglycemia, prediabetes, or T2DM.

## Methods

### Participant recruitment and disposition

Patients were recruited in the Abu Dhabi Sleep Apnea (ADSA) research project (N = 674), a study of sleep apnea prevalence at Imperial College London Diabetes Centre (ICLDC), an outpatient diabetes and endocrinology institute in Abu Dhabi, United Arab Emirates (UAE). Written informed consent for the involvement in the sleep apnea study was obtained from all research participants, and another for the use of anonymized medical data for research purposes were derived from all patients at the time of the first visit to the center. The ADSA study was approved by the ICLDC Research Ethics Committee and followed the Declaration of Helsinki, 1996. NC was measured at the level of the thyroid cartilage, and collar size was recorded; participants completed a questionnaire including the STOP-BANG (Snoring, Tiredness, Observed apnea, blood Pressure, BMI, Age, NC, and Gender) criteria for sleep apnea. Participant information, including BMI, blood pressure, diabetes status, smoking status, medications, and contemporaneous HbA1c, full blood count, lipid profile, and liver function tests, was retrieved from the electronic medical records. Diabetes and smoking status were derived from the individual patient records. No participant reported alcohol use. Medication compliance was assessed based on the prescriptions of physicians and/or clinic notes. HbA1c was measured using the VARIANT II system (Bio-Rad). Biochemical parameters were assessed using the Cobas platform (Roche).

For the purposes of statistical analysis, individuals with secondary diabetes, type 1 diabetes, or MODY were excluded (n = 22). Individuals diagnosed with impaired glucose tolerance, impaired fasting glucose, or previous gestational diabetes were considered to have prediabetes. Individuals without a record of parameters for the calculation of cardiovascular risk and NAFLD fibrosis score were excluded from further analysis (n = 105). Four individuals had serological evidence of active viral hepatitis infection and were therefore excluded from the analysis. In total, 547 individuals were included in the statistical analysis. Baseline characteristics of the included participants are presented in **Table 1**.

**Table 1:** Baseline characteristics of participants included in statistical analysis. Values are presented as mean ± standard deviation.

Grouping	NGT	Pre	T2DM
Number	113	108	326
Female (%)	74.3%	51.9%	42.6%
Age	35.4 ± 10.3	44.5 ± 10.6	50.3 ± 9.2
BMI	28.5 ± 6.1	30.3 ± 5.4	32.1 ± 5.9
NC	35.1 ± 3.1	37.4 ± 3.2	38.9 ± 3.3
sBP (mmHg)	116.9 ± 13.6	123.6 ± 15.8	128.3 ± 17.7
dBP (mmHg)	68.7 ± 9.8	73.9 ± 11.9	75.5 ± 10.3
HbA1c (%)	5.1 ± 0.4	5.6 ± 0.8	7 ± 1.5
ALT (IU/ml)	19.8 ± 12.9	25.9 ± 13.9	27.2 ± 15.5
AST (IU/ml)	18.4 ± 6.4	20.4 ± 8.5	20.1 ± 9.5
HDL (mmol/L)	1.5 ± 0.4	1.4 ± 0.4	1.2 ± 0.3
LDL (mmol/L)	3 ± 0.8	3 ± 0.9	2.6 ± 0.9
TG (mmol/L)	1.1 ± 0.6	1.3 ± 0.8	1.7 ± 1
FLI ≥60	14 (35.9%)	37 (48.1%)	221 (70.2%)
HSI ≥36	67 (59.3%)	87 (80.6%)	305 (93.6%)
STOP BANG Score	1.8 ± 1.5	2.9 ± 1.8	3.6 ± 1.8

NGT, normal glucose tolerance; Pre, prediabetes; T2DM, type 2 diabetes mellitus; BMI, body mass index; NC, neck circumference; sBP, systolic blood pressure; dBP, diastolic blood pressure; HbA1c, glycated hemoglobin; ALT, alanine transaminase; AST, aspartate aminotransferase; HDL, high-density lipoprotein; LDL, low-density lipoprotein; TG, triglycerides.

### Statistical analysis

Data are presented as mean ± standard deviation. Statistical analysis was performed using the R language for Statistical Computing version 4.1.3 with the *survival* and *icenReg* packages. NAFLD Fibrosis Score was calculated as (−1.675 + 0.037 ∗ Age (years) + 0.094 ∗ BMI + 1.13 ∗ (presence of prediabetes or diabetes) + 0.99 ∗ (AST/ALT) – 0.013 ∗ Platelets (10^9^/L) – 0.66 ∗ Albumin (g/dl)). The Fatty Liver Index (FLI) was calculated as (FLI coefficient/(1 + FLI coefficient) ∗ 100) where the FLI coefficient is exp(0.953 ∗ ln(Triglycerides) + 0.139 ∗ BMI + 0.718 ∗ ln(GGT (IU/L)) + 0.053 ∗ Waist circumference − 15.745), with an FLI score of ≥60 indicating a high risk of fatty liver disease. The Hepatic Steatosis Index (HSI) was calculated as (8 ∗ ALT/AST) ∗ BMI + 2 ∗ (if female) + 2 ∗ (if Type 2 Diabetes), with an HSI score of ≥36 indicating high risk for fatty liver disease. The FIB4 score was calculated as (Age (years) ∗ AST (IU/L))/(Platelets (10^9^/L) ∗ sqrt(ALT (IU/L))). Ten-year cardiovascular risk was calculated using the Pooled Cohort Equations for White individuals (24). Linear regression models were used for adjustment for covariates in the analysis of the association between neck circumference and study outcome measures; logistic regression models were used to adjust for covariates in comparisons between glycemic status groups. Cox proportional hazards models were used for prospective analysis of progression to type 2 diabetes in individuals with prediabetes or diabetes at enrolment, while Cox regression with adjustment for left censoring according to the methods of Wei Pan (1999) using the specific implementation by Anderson-Bergman (2020) was used to investigate the longitudinal relationship between neck circumference and ALT. Derangement of ALT was defined as ≥33 IU/L in males and ≥25 IU/L in females according to ACG criteria (25) while interval to progression to type 2 diabetes was defined as the time in years between enrolment and first recorded HbA1c ≥6.5%, clinical diagnosis of type 2 diabetes, or prescription of hypoglycemic medication for type 2 diabetes. Significance was assessed at the level of p <0.05; no correction was made for multiple comparisons.

## Results

### Relationships between NC and glycemic status

**Figure 1:**
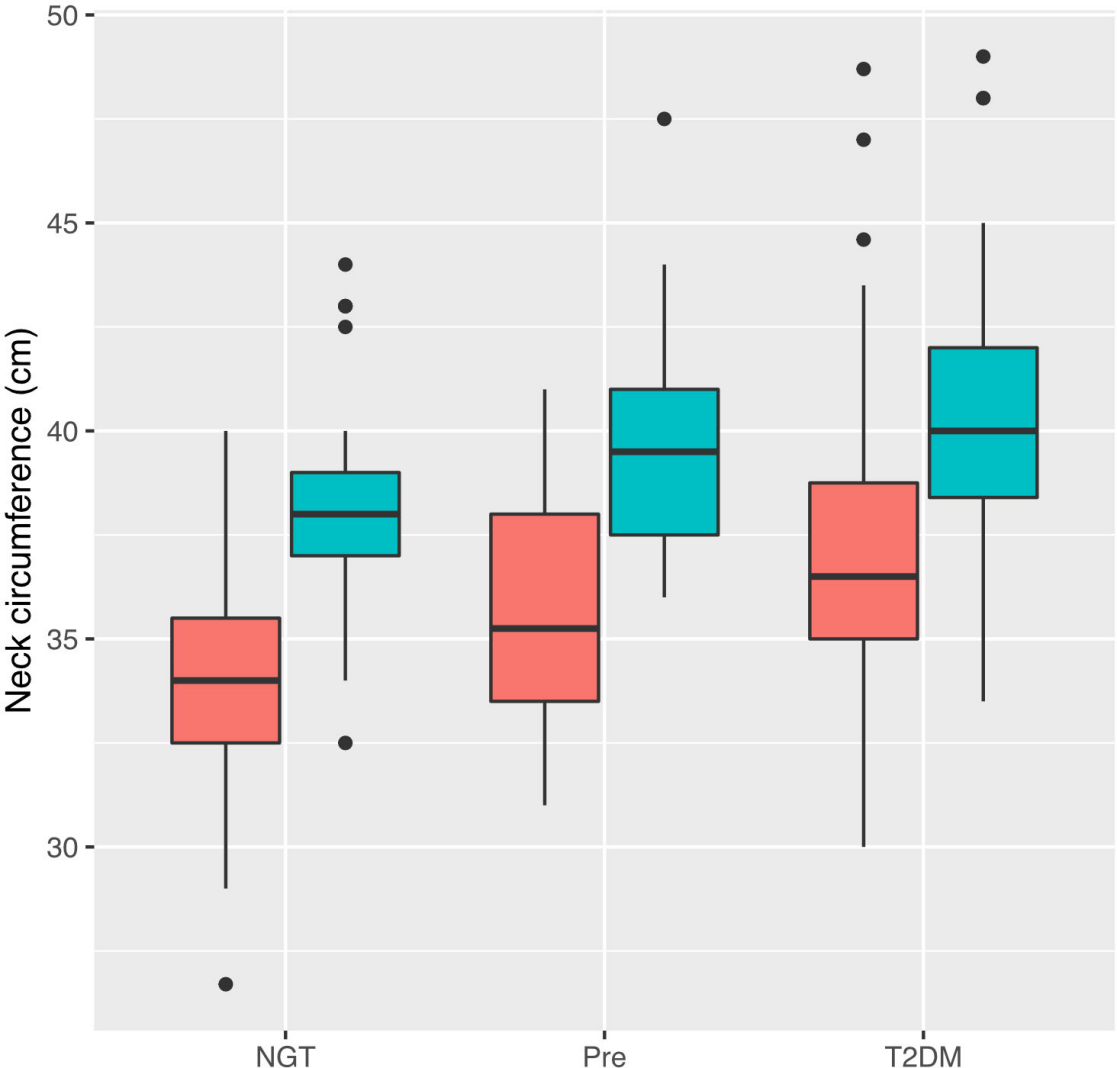
Relationship between neck circumference and glycemic status stratified by sex. Tukey plot represents median, interquartile range (IQR) and IQR ± 1.5 * IQR. Male participants are represented by blue and female participants by red. NGT, normal glucose tolerance; Pre, prediabetes; T2DM, Type 2 diabetes mellitus.

The mean neck circumference at enrollment was 35.1 ± 3.1 cm in individuals with normal glucose tolerance, 37.4 ± 3.2 cm in individuals with prediabetes, and 38.9 ± 3.3 cm in people with type 2 diabetes. In each of these groups, NC was significantly greater in men than in women (38.3 ± 2.8 cf 34 ± 2.3, p <0.0001; 39.5 ± 2.4 cf 35.5 ± 2.6, p <0.0001; 40.2 ± 2.8 cf 37.1 ± 3, p <0.0001, respectively). After adjusting for age, sex, and BMI in logistic regression, neck circumference was significantly increased in people with prediabetes compared with those with normal glucose tolerance (difference 0.65 ± 0.3 cm, p = p = 0.0331). In the same analysis, type 2 diabetes was significantly associated with increased neck circumference compared with the normal glucose tolerance group (difference 1.07 ± 0.28 cm, p = 0.0002). The relationships between NC and sex-stratified glycemic status are presented in **Figure 1**.

Among 171 individuals with normal glucose tolerance or prediabetes at enrollment and subsequent HbA1c measurement (median follow-up 4.6 (2.3–6.5) years), four with NGT and 26 with prediabetes at enrollment progressed to type 2 diabetes. The neck circumference was significantly and independently associated with an increased hazard of subsequent progression to type 2 diabetes, adjusted for BMI (neck circumference: hazard ratio 1.141, 95% CI 1.004–1.296, p = 0.043, BMI: hazard ratio 1.043, 95% CI 0.977–1.114, p = 0.207). NC was not a significant predictor when further adjusted for age, sex, or prediabetes, although this analysis was limited by the small number of available endpoints for each covariate. NC did not significantly predict incident retinal or renal microvascular complications either in univariate analysis or when adjusted for age, sex, HbA1c, blood pressure, smoking status, and BMI over a median follow-up period of 4.8 (IQR 2.3–6.1) years.

### Relationships between NC and hazard of liver disease

**Figure 2:**
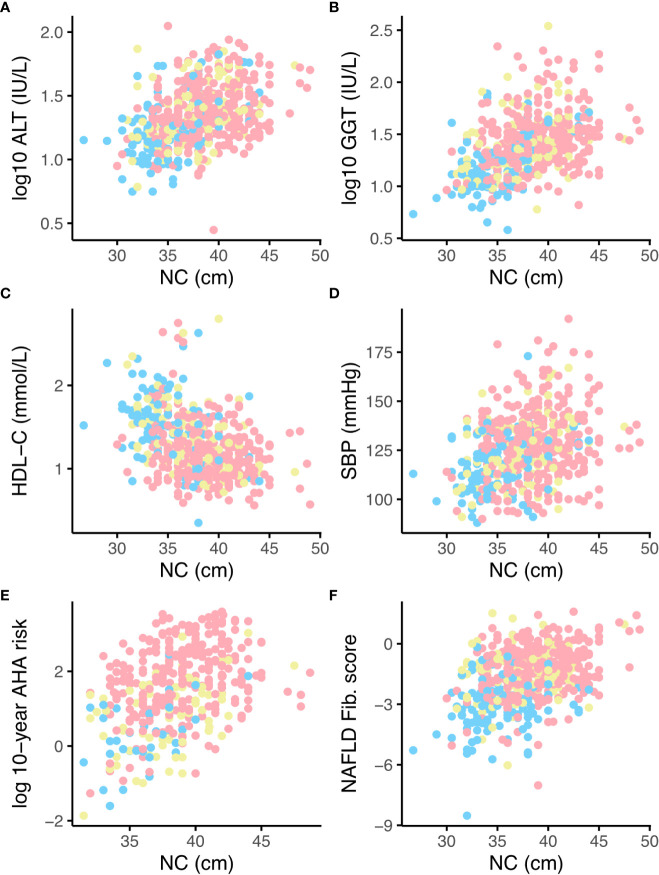
Relationships between neck circumference (NC) and liver function tests, lipid profile, blood pressure, AHA 10-year cardiovascular risk and NAFLD Fibrosis score, stratified by glycaemic status. NGT = blue, Prediabetes = yellow, Type 2 diabetes = pink. Panels **(A)** log-transformed ALT, **(B)** log-transformed GGT, **(C)** HDL-cholesterol (HDL-C), **(D)** systolic blood pressure **(E)** log-transformed 10-year cardiovascular risk assessed by AHA pooled-cohort equations, **(F)** NAFLD fibrosis score. ALT, alanine transaminase; GGT, gamma-glutamyl transferase; SBP, systolic blood pressure; AHA, American Heart Association; NAFLD, non-alcoholic fatty liver disease.

The relationship between neck circumference and log-transformed serum ALT was approximately linear (r = 0.407, p <0.0001, Pearson), as illustrated in **Figure 2A**. Adjusting for body mass index, sex, and glycemic status, a 1-cm increase in NC was significantly and independently associated with a 1.04 ± 1.01 U/L (p <0.0001) increase in ALT and a 1.05 ± 1.01 U/L (p <0.0001) increase in gamma-GT (GGT), illustrated in **Figure 2B**. Male sex and type 2 diabetes diagnosis were significantly and positively associated with ALT, while male sex, prediabetes, and type 2 diabetes were significantly and positively associated with GGT, consistent with previous reports (25, 26).

In univariate linear regression, a 1-cm increase in NC was significantly associated with a 0.16 ± 0.02 increase in NAFLD fibrosis score (p <0.0001, see also **Figure 2F**); this analysis was not adjusted for age or diabetes status since these are components of the risk score, but did remain significant when adjusted for sex. The univariate association between NAFLD fibrosis score and neck circumference remained significant in 272 individuals with an FLI score of ≥60, suggestive of NAFLD, at recruitment (0.08 ± 0.02 increase in NAFLD fibrosis score per 1-cm increase in NC, p = 0.0004). A 1-cm increase in NC was also significantly associated with a 2.72% increase in FIB4 score (p <0.0001) in the group as a whole and a 2.11% increase in FIB4 score in individuals with an FLI suggestive of NAFLD at enrollment (p = 0.0287). These analyses are limited by missing data for waist circumference, and therefore the HSI NAFLD risk score was also calculated. In 459 individuals with an HSI score ≥36, suggestive of the presence of NAFLD, a 1% increase in NC was associated with a 0.12 ± 0.02 increase in NAFLD fibrosis score (p <0.0001) and a 2.55% increase in FIB4 score (p = 0.0002).

**Figure 3:**
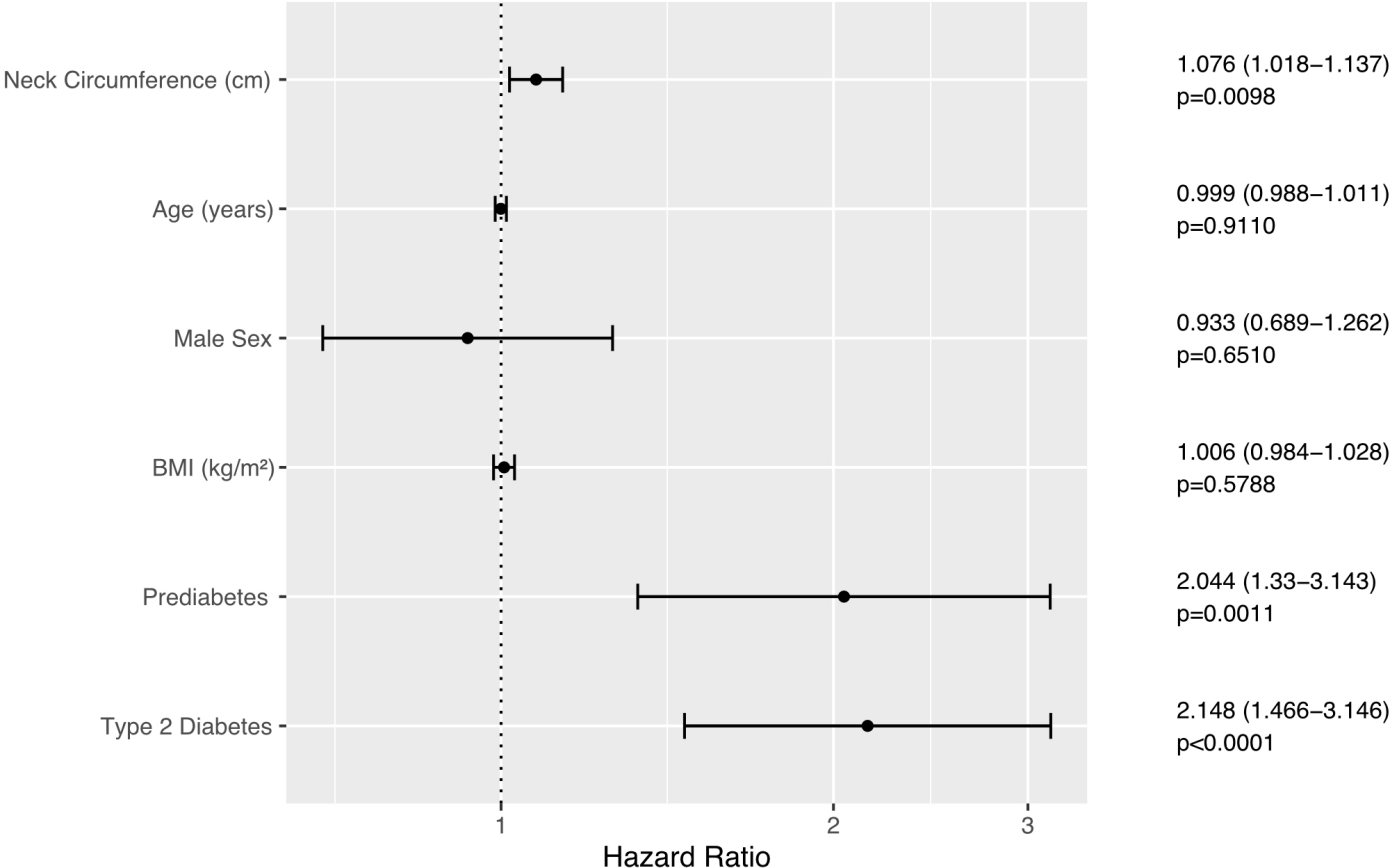
Forest plot illustrating Cox Proportional Hazards model of onset of elevation of ALT (≥33 in males, ≥25 in females). Error bars represent the 95% confidence interval of the hazard ratio.

**Figure 4:**
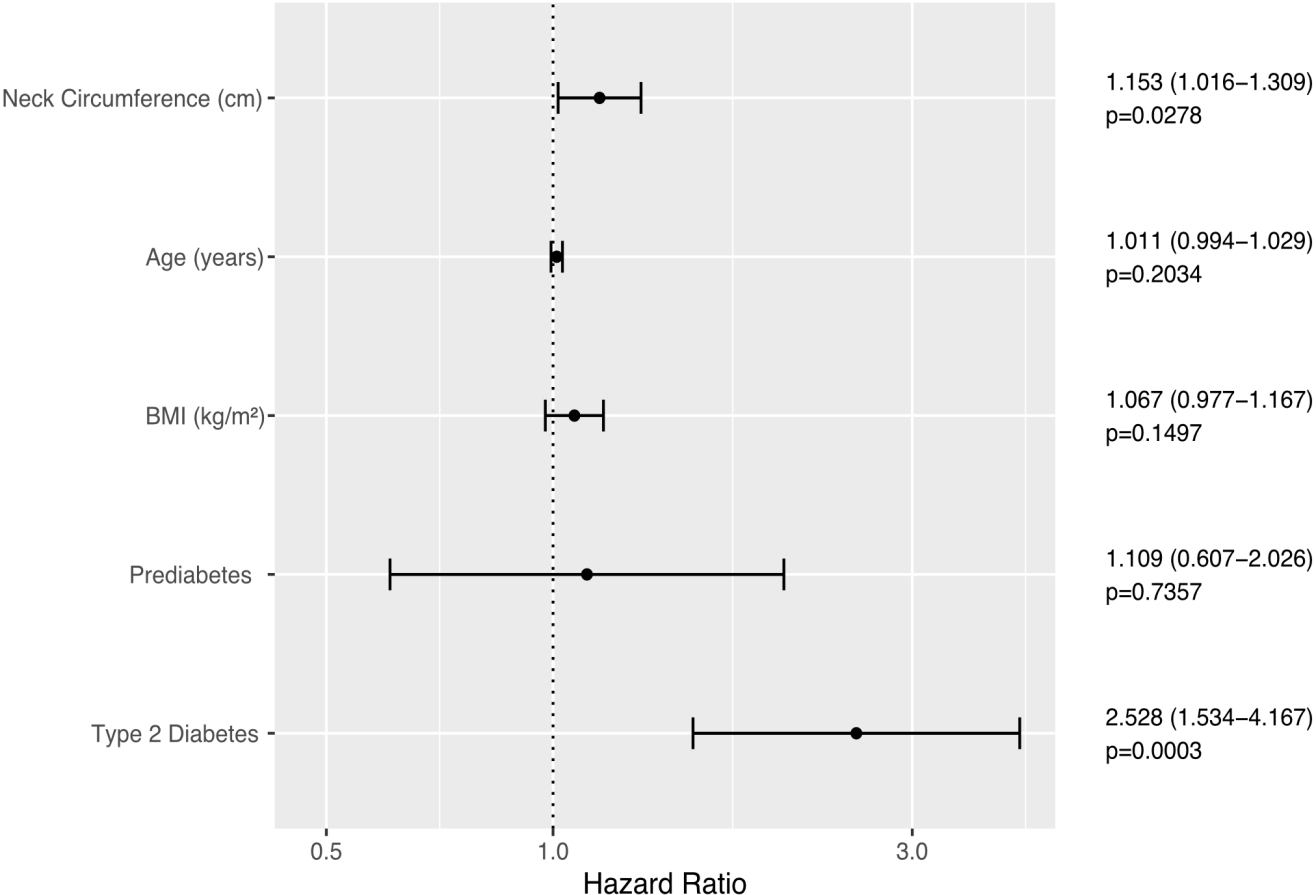
Forest plot illustrating Cox Proportional Hazards model of time to first FLI ≥60 in individuals with FLI <60 at enrolment. Error bars represent the 95% confidence interval of the hazard ratio.

NC was positively and independently associated with incident elevation of ALT in Cox proportional hazards regression adjusted for age, sex, BMI, and glycemic status (hazard ratio 1.076 (95% CI 1.015–1.14, p = 0.0131) per 1-cm increase in NC); both prediabetes and type 2 diabetes were also significant predictors (hazard ratios 2.044 (95% CI 1.377–3.034), 2.148 (95% CI 1.419–3.25), respectively, see also **Figure 3**). In a subset of 395 participants with sufficient data, NC remained significantly associated with new derangement of ALT when adjusted for waist-hip ratio instead of BMI. Stratifying for sex and adjusting for age, BMI, and glycemic status, NC was also significantly and independently associated with progression to FLI ≥60 in individuals with low FLI-assessed risk of NAFLD at enrollment (hazard ratio 1.153 (95% CI 1.019–1.304), p = 0.0239, see also **Figure 4**). During the follow-up period, 53 participants (47 with HSI ≥36 at enrollment) progressed to a NAFLD Fibrosis Score of ≥0.676, while 12 participants (11 with HSI ≥36 at enrollment) progressed to a FIB-4 score of ≥2.67, both cut-offs for high risk of advanced fibrosis. NC predicted progression to the high NAFLD Fibrosis Score cut-off of ≥0.676 during the follow-up period in univariate and analysis and when adjusted for sex (hazard ratio 1.076, p <0.01. However, NC did not significantly predict progression to NAFLD Fibrosis Score ≥0.676 when adjusted for BMI or diabetes status, or in univariate analysis when limited to patients with an HSI ≥36. In univariate or multivariate analysis, NC did not predict progression to the FIB-4 cut-off of ≥2.67, either in the entire study population or in individuals with HSI ≥36, although this analysis was limited by the small number of endpoints.

### Relationship between NC and cardiovascular risk factors

A 1-cm increase in NC was significantly associated with an increase in systolic blood pressure blood pressure of 0.65 ± 0.24 mmHg (p = 0.0066), adjusted for age, sex, glycemic status, and antihypertensive use (see also **Figure 2D**), and a reduction in HDL-C of 0.03 ± 0.01 mmol/L (p <0.0001) adjusted for age, sex, glycemic status, and BMI. In 384 participants aged ≥40 years, NC was associated with an increase in the 10-year AHA cardiovascular risk score of 1.15 ± 1.02% (p <0.0001) per 1-cm increase (**Figure 2E**). NC was negatively associated with HDL (**Figure 2C**) and positively associated with a 0.1 ± 0.02 increase in TC : HDL (p <0.0001) adjusted for age, sex, glycemic status, and statin use, and hence, along with systolic blood pressure, two of the modifiable risk factors included in the Qrisk lifetime cardiovascular risk equation. Although NC was not significantly associated with incident macrovascular disease (new records of ICD-10 codes I20, I21, I24, I25, I63, I70, and I73) in a longitudinal Cox proportional hazards analysis adjusted for age, sex, glycemic status, blood pressure, LDL-C, and smoking status, although since ICLDC is not primarily a cardiology center and these diagnostic codes were therefore based on patient recall, this analysis may have been limited by under-reporting of events.

## Discussion

Metabolic anomalies arising from obesity have been attributed to visceral or upper body subcutaneous fat deposits, predominantly elevated levels of free fatty acids (FFA) mediated by insulin resistance (27–29), The increase in FFA concentrations is positively associated with interrelated MetS components—abdominal obesity, hypertension, dysglycemia, and hyperlipidemia, which share underlying pathways including inflammation, the final common pathway (29–32). MetS is the clustering of these components, and its global prevalence has been reported to be 20%–25% in adults and up to 19% in children with type 1 diabetes (33). The Metabolic Syndrome, dysglycemia, and NAFLD are all highly prevalent among Emirati nationals in the UAE, at 33.6%, 40.0%, and 34.7%, respectively (34–36). Assessments of prevalence are, however, limited by the lack of a unified MetS definition since multiple criteria from the World Health Organization (WHO), National Cholesterol Education Program (NCEP), International Diabetes Federation (IDF), American Association of Clinical Endocrinologists (AACE), and European Group of Insulin Resistance (EGIR) are all used in clinical research (37).

The association of neck adiposity with MetS has been recognized since the early 1950s (38). Ben-Noun et al. reported positive correlations between NC, CVD risk factors, and blood pressure, as well as corresponding changes in both (39–41). NC among participants in the Framingham Heart Study was associated with CVD risk factors after adjusting for BMI and VAT (28). In keeping with these previous findings, we found positive associations between NC and sBP, 10-year cardiovascular risk, and modifiable lifetime cardiovascular risk factors. Compartmentalization of neck fat accumulation in a study by Torriani et al. found that neck adipose tissue (NAT), most notably posterior cervical NAT (NATpost) and subcutaneous NAT (NATsc), was associated with CVD risk factors and MetS, and more prominently among women (32). The higher association in women compared to men was similarly observed in other studies (28) and was partly ascribed to higher upper body FFA in women (42). These findings and the results of this study, particularly the positive, significant, association of male sex and T2DM with ALT and GGT, require further research.

A systematic review and meta-analysis performed in 2017 (43) found that NC is an accurate tool for assessing overweight and obesity in both males and females and across different age groups, although cut-off points for different populations were suggested. This was also the recommendation of a systematic review of the association between NC with cardiometabolic risk in adolescents (23), which found the relatedness of NC with BMI, WC, and MetS. In contrast, a similar systematic review and meta-analysis in an adult population did not find an association of NC with MetS but only with BMI, WC, hypertension, fasting blood sugar (FBS), total cholesterol (TC), LDL-C, sBP, diastolic blood pressure (DBP), and low HDL-C concentrations. However, heterogeneity between studies was high, therefore findings were advised to be taken with caution (17). In this study, although NC was positively associated with several MetS risk factors, it was not found to be so with new retinopathy, maculopathy, or microalbuminuria. A recent study by Sobhani et al. found that BMI was a consistent predictor of triglycerides and increased hepatic enzymes, although the relevance of NC was not indicated (44). Other recent studies have reported that obesity is involved with microvascular disease progression, including retinopathy in T2D (45, 46). These reports may again underscore the need for additional studies and population-specific NC cut-offs for appropriate correlations with obesity, MetS, and other related conditions such as dysglycemia and fatty liver.

A more recent systematic review, authored by a group from the UAE, reported a weak association between NC and BMI (47). In another study in 2021 with Emirati adults as participants, results similarly showed a poor correlation between NC and BMI, WC, and WHR (48). However, a study on adult females in the UAE in 2015 reported a significant positive relationship between NC and obesity (49). Although this may be related to having female participants as previously discussed, conflicting results, including several outcomes of the current study again, signify the need for further investigation of NC along with other obesity anthropometric measures in this population and region.

NC is correlated with ultrasound-assessed liver fat content in adult non-obese (50–53) and pediatric and adolescent obese (54) populations. NC was positively associated with intensity of histologically assessed liver steatohepatitis but not with the presence of steatosis or presence of fibrosis in a study of 119 predominantly female, non-diabetic obese patients undergoing bariatric surgery (55). NC was not significantly associated with the intensity of steatohepatitis in multivariate regression with BMI and WC, although each measure was equally weakly associated (R = 0.2 for all) in univariate analysis and the number of endpoints analyzed was relatively small given the expected collinearity between these variables (55). Despite a large proportion of our patients with an HSI suggestive of hepatic steatosis at enrollment, a surprisingly small number of individuals went on to be predicted to be at high risk of fibrosis according to the FIB-4 score during follow-up. The FIB-4 score has been observed to have an unexpectedly high false-negative rate in patients with type 2 diabetes, particularly at the intermediate-risk cut-off of 1.3 (56, 57). The FIB-4 score is also reported to have a high false negative rate in individuals under the age of 35 years and an increased false positive rate in individuals over 65, partially attributed to the inclusion of age in the FIB-4 calculation (57).

### Study strengths and limitations

The study strengths include the number of individuals in whom NC was measured and the consistent quality of data for follow-up. The NC measurements were taken as part of a research protocol and in a single centre, increasing the reliability of the source data. Our dataset allowed us to explore associations of NC with subsequent incidence of adverse metabolic characteristics, where much of the existing literature examines correlational relationships with prevalent disease. Additionally, this is a population-specific investigation of NC as a potential assessment tool for obesity and related disorders. Study limitations include the retrospective nature of a part of the study, which relied on the retrieval of electronic medical records. This study is also based on clinical data and may therefore be subject to reporting bias for clinical endpoints. The measurement of WC and HC, although performed by trained clinical staff, may have been inaccurate because of challenges in locating anatomical landmarks. A prospective study with the inclusion of comprehensive, well-defined parameters would be valuable.

## Conclusions

In an Emirati cohort, NC was associated with dysglycemia and markers of MetS. Our data suggest that NC could play a role in identifying people with prediabetes or diabetes who are at increased risk of NAFLD but do not support an association between NC and incident liver fibrosis.

## Data availability statement

The raw data supporting the conclusions of this article will be made available by the authors, without undue reservation.

## Ethics statement

The studies involving human participants were reviewed and approved by the Research Ethics Committee, Imperial College London Diabetes Centre. The patients/participants provided their written informed consent to participate in this study.

## Author contributions

EF: study design, data acquisition, and manuscript writing. AB: study design, statistical analyses, and manuscript writing. NL: data interpretation and manuscript editing. All authors listed have made a substantial, direct, and intellectual contribution to the work and approved it for publication.

## Conflict of interest

The authors declare that the research was conducted in the absence of any commercial or financial relationships that could be construed as a potential conflict of interest.

## Publisher’s note

All claims expressed in this article are solely those of the authors and do not necessarily represent those of their affiliated organizations, or those of the publisher, the editors and the reviewers. Any product that may be evaluated in this article, or claim that may be made by its manufacturer, is not guaranteed or endorsed by the publisher.
